# Impact of exercise training and diet therapy on the physical fitness, quality of life, and immune response of people living with HIV/AIDS: a randomized controlled trial

**DOI:** 10.1186/s12889-024-17700-0

**Published:** 2024-03-06

**Authors:** Xin-Min Qin, Robert Allan, Ji-Young Park, Sung-Hoon Kim, Chang-Hwa Joo

**Affiliations:** 1 Department of Sport Science, Kangwon National University, 1 Gangwondaehakgil, Chuncheon-si, Gangwon-do 24341 Republic of Korea; 2 Department of Smart Health Science and Technology Convergence, Kangwon National University, 1 Gangwondaehakgil, Chuncheon-si, Gangwon-do 24341 Republic of Korea; 3 Research Centre for Applied Sport, University of Central Lancashire, Lancashire, UK; 4 Department of Physical Education, Yonsei University, 50 Yonsei-ro, Seodaemun-gu, Seoul, 03722 Republic of Korea

**Keywords:** Exercise, Diet therapy, Training, Physical fitness, Immune response

## Abstract

**Background:**

Exercise and dietary nutrition are considered crucial in human immunodeficiency virus (HIV)/ acquired immunodeficiency syndrome (AIDS) treatment protocols and people living with HIV/AIDS (PLWHA) rehabilitation care. However, there is no well-studied research evaluating the effects of combined interventions on the fitness and immune systems of PLWHA. Therefore, this study aimed to analyze the effects of exercise and dietary intervention on physical fitness, quality of life and immune response in PLWHA.

**Methods:**

This was an experimental study, with a sample of 25 male PLWHA divided into two groups: the intervention group (IG: 12 participants) and the control group (CG: 13 participants). All participants have not had any exercise habits and nutritional supplements in the past six months. The participants in the IG completed 45 min of exercise (60-80% HRmax) 4 times per week for 4 weeks. The exercise was in the form of brisk walking or running. They were also given a nutritional dietary supplement 3 times a day for 4 weeks. The 13 individuals in the CG continued their normal daily life (physical activity and diet). The following parameters were evaluated before and after the intervention: body composition, physical fitness, immune response, quality of life (QoL), stress, dietary behavior, dietary habits, exercise motivation, and physical self-efficacy.

**Results:**

The significant changes were observed in burnout of stress variables and physical efficiency index (PEI) of physical fitness in the IG (*p* =.023). Moreover, in the saliva samples, sal-T levels significantly increased only after the intervention in the IG (*p* =.012). Additionally, regarding the analysis of the interaction (group × time), there was a significant improvement in the reaction speed (*p* =.001) and grip strength (left: *p* =.002, right: *p* =.030) and a significant difference in physical satisfaction in QoL (*p* =.001), stress burnout (*p* =.043), self-confidence in physical efficacy (*p* =.045), external display (*p* =.008), and fulfillment (*p* =.047) in exercise motivation. Moreover, the significant effect of the intervention on emotional eating in dietary behavior was shown in the comparison of the IG before and after intervention (*p* =.001) and in the comparison of the IG group with the CG after the experiment (*p* =.013). However, there was no significant effect of time or interaction between the condition and time on body composition.

**Conclusions:**

In conclusion, exercise training and diet therapy caused changes in physical fitness and Sal-T levels, which had positive effects on the health promotion of PLWHA.

## Background

Acquired Immune Deficiency Syndrome (AIDS) is caused by human immunodeficiency virus (HIV) infection [1], which targets the immune system and weakens the human body’s defense system against infections and certain types of cancer [2]. A total of 39 million people were infected and 630,000 people were killed worldwide by HIV in 2022 [3]. The number of people infected with HIV is increasing every year. According to statistics from the Korea disease control and prevention agency, 1,066 new cases of HIV infection were registered in 2022, an increase of 9.3% from 2021 to 4.9% from 2020. Among them, 66.4% were aged between 20 and 30 years, and 99.1% were infected through sexual contact [4]. Infected people tend to hide the fact that they are infected due to social stigmatization, and the actual number of infected people is likely higher than the statistical figure. In the past, HIV infection was a terrifying infectious disease that resulted in death, but antiretroviral treatment (ART) has prolonged the lifespan of infected people [5]. Although ART promotes the growth of CD4 + cells and inhibits the spread of the virus, it can also cause side effects [6]. People living with HIV/AIDS (PLWHA) were restricted in their social life because of health problems before the application of ART, but these restrictions were relatively reduced by health improvement through ART treatment.

HIV can affect the psychological health of a person through a combination of causes. The challenges faced by people living with HIV/AIDS (PLWHA) during daily life, including physical symptoms, psychological distress, and social isolation, significantly influence their quality of life (QoL) [7, 8]. Low QoL may be related to negative emotions, such as depression and anxiety, which can lead to poor physical health status by reducing immune function. Indeed, immune system responses, such as disease-induced inflammation, can negatively influence psychological emotions [9]. Concurrently, psychological stress is associated with the suppression of cellular and humoral immunity [10]. The psychological adjustment in PLWHA is influenced by adjustment methods based on perceived deterioration and adherence treatment through coping strategies [11]. Therefore, appropriate treatment methods should be applied along with drug treatment to maintain the physical and psychological health of PLWHA.

Exercise training has been used to manage the signs and symptoms of chronic diseases, and is widely used in health promotion and rehabilitation [12]. Numerous studies have shown that exercise improves QoL, physical fitness [13], motor skills [14] and cardiovascular fitness [15]. Furthermore, exercise training regulates and enhances the immune system. According to the literature, it has a positive effect on the immune function [16]; a 12-week aerobic exercise research also reported a reduction in inflammation with exercise [17]. It also plays a positive role in the prevention and treatment of diseases and is considered a supplement to the medical care and treatment of PLWHA [18]. Additionally, PLWHA recognize the benefits of exercise activities in promoting QoL and immunity and believe that exercise activities should be of higher priority in their lives [19]. A recent study found that the exercise training program provided successful conservative treatment for some HIV comorbidities and side effects of therapy [20]. Exercise intensity, duration, and volume can affect the redistribution of exercise-related immune cells in the circulatory system [21]. Moderate-intensity exercise is recommended for the general public and for PLWHA to enhance health [22, 23].

It is well-known that proper nutrition is essential for physical health in the daily life of individuals. Several studies have indicated that basal metabolism increases during the asymptomatic or symptomatic period of HIV infection due to the cascade of inflammatory responses caused by the virus [24–26]. PLWHA are prone to decreased nutrient absorption due to intestinal damage, and their food intake is also reduced due to vomiting and swallowing pain, which aggravates the decline in QoL and immune function [27]. ART drug side effects and long-term inadequate food sources containing calcium, vitamin D, magnesium, and phosphorus increase the risk of bone loss in PLWHA [25, 28, 29]. Dietary nutritional intervention measures can help PLWHA effectively avoid the negative effects of drugs and improve their immune metabolism and the therapeutic effects of ART [27]. Therefore, PLWHA require additional energy compensation to maintain a stable nutritional status. However, they tend not to have a well-balanced diet due to unstable jobs and low-income levels in South Korea.

In addition, some investigations have validated the effect of a combination of exercise training and nutritional supplementation on the prevention and treatment of diseases. Preventing a decline in the physical and nutritional status can have a significant effect on the care continuum [30]. High levels of physical activity and good dietary habits have also been noted to moderately affect the sleep quality [31]. Moreover, exercise training and low-carbohydrate and high-protein diet may improve the mental health in women with obesity [32]. Exercise and nutrition are also considered crucial in treatment protocols and rehabilitation care for PLWHA. However, although there are several studies that separately evaluate the effect of exercise and nutrition in PLWHA, there is no thorough research evaluating a combination of the two in this population. In this study, we aimed to explore the effects of a combined intervention of exercise and diet in PLWHA.

## Methods

### Aim and study procedures

**Fig. 1:**
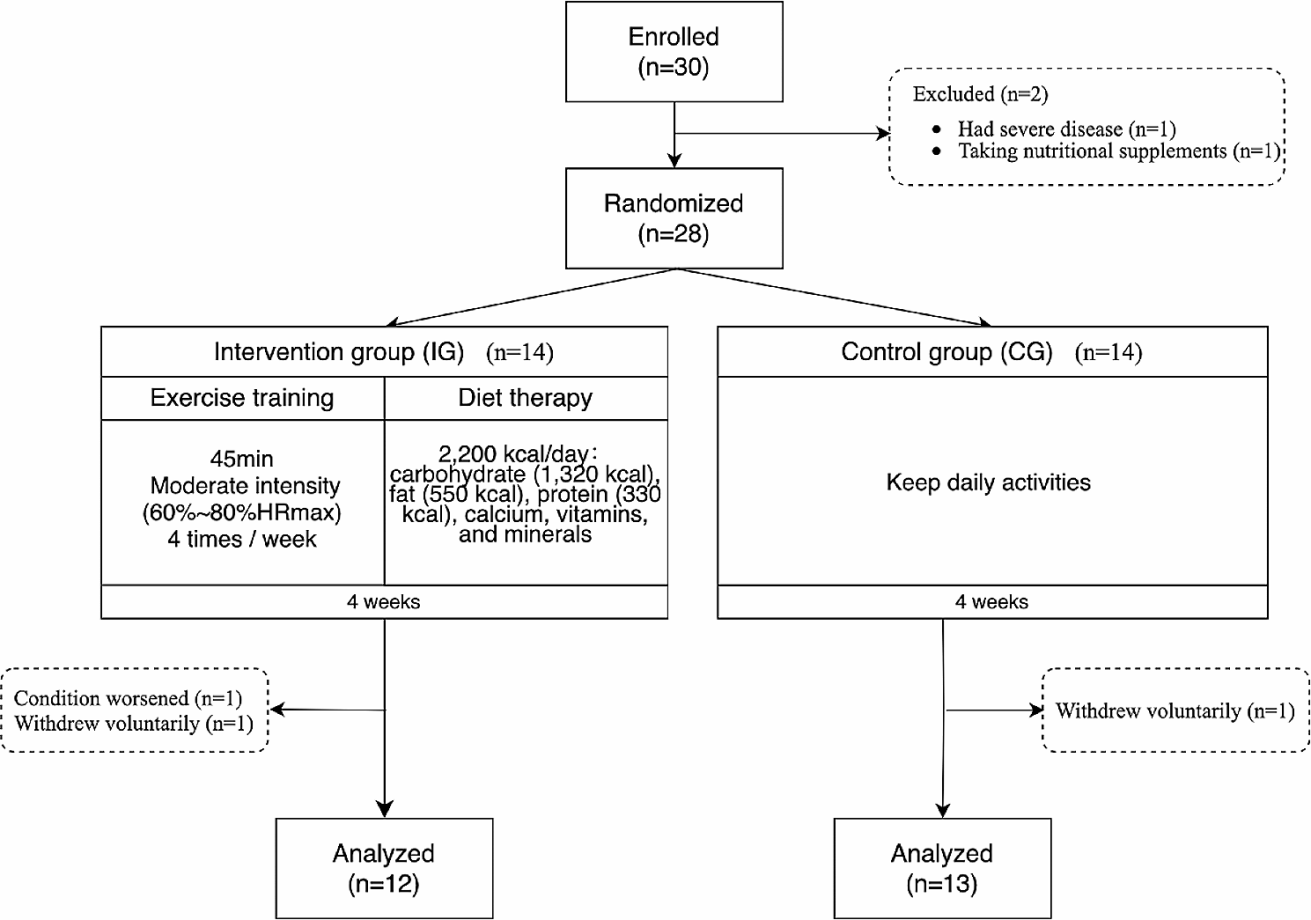
Experimental group assignment

This study aimed to analyze the effects of exercise and dietary intervention on body composition, physical fitness, immune response and QoL in PLWHA. This was a randomized controlled trial involving control or exercise training with nutritional supplement interventions (Fig. 1). After arriving at the collection center, the participants changed into uniform sportswear and rested for 10 min. After being fully rested, data were collected in the following order: (1) heart rate (HR) and blood pressure at rest; (2) saliva collection; (3) body composition; and (4) questionnaire collection and physical fitness tests. The data was collected twice: at baseline and at the end of the experiment, at 10–12 am each time. The final data collection period was at least 24 h after the last training session. The content of each data acquisition and requirement remained the same. The investigator in charge of the data acquisition underwent the same procedure to avoid inter-rater errors.

A detailed selection process of the exercise intensity and duration was presented in the earlier publication of the study results [23]. Moderate-intensity (60-80% HRmax) exercise was used for exercise training. The participants completed the 45min exercise 4 times per week for 4 weeks. The exercise was in the form of brisk walking or running. The exercise was performed on a sports field or park near the participants’ homes. The participants controlled the exercise intensity by observing their heart rates using a monitoring device (Polar Wear Link®; Polar Electro, Kempele, Finland). The exercise included four stages: (1) 3-min warm-up, (2) 3∼4 min to reach the target heart rate (THR) (60-80% HRmax), (3) 45-min exercise with THR, and (4) 3 min to cool down.

To determine the usual nutritional intake level of the participants, an analysis was conducted using a self-written nutrition questionnaire (Diet Record Sheet; DRS) for two weeks before the experiment. As a result of the analysis, both groups showed lower average energy intake of all energy sources except carbohydrates when compared to the “2020 Korean Nutrient Intake Standards” announced by the Ministry of Health and Welfare [33]. Therefore, a dietary supplement of carbohydrate, protein, fat, calcium, vitamins, and minerals was provided to the IG group according to consideration of the “2020 Korean Nutrient Intake Standards” in this study. The amount of food was calibrated to represent approximately 2,200 kcal/day according to age. The total energy requirement was 1,320 kcal/day through carbohydrates, 550 kcal/day through fats, and 330 kcal/day through proteins; we also provided 120 g of vegetable salad and 200 g of milk daily for calcium, vitamins, and minerals. Diet therapy interventions were monitored and recorded using the DRS and dieticians. The participants recorded their diet situation (date, time, and calories) daily. Dieticians monitored the diet therapy through telephone follow-ups to examine the dietary diaries of each participant every week. All participants in the CG were instructed to continue their daily activities (physical activity and diet) during the experimental period. Daily physical activity was maintained without physical exercise, training, daily eating habits, or nutritional supplements during the study period.

### Participants

Thirty male PLWHA who had been screened for HIV with positive results were recruited through printed posters, social media, and the Korean Association for AIDS Prevention Center. Participants who met any of the following criteria were excluded: (1) had severe diseases (e.g., cardiovascular disease, severe depression, and cancer), (2) had communication impairments; (3) had cognitive impairment, (4) were hospitalized or living in a nursing home, (5) participated in other studies, (6) underwent physical activity, (7) were receiving nutritional supplements at the time of recruitment, (8) their condition worsened, or (9) they withdrew voluntarily. Two participants were excluded because they were unable to complete the test due to leg injury and another was excluded because he was taking nutritional supplements at the time of recruitment. Twenty-eight tags with A and B (14 each) were placed in the same envelope, and participants randomly chose one. Those who selected the tags with A were included in the intervention (exercise and diet therapy) group (IG, *n* = 14), and those who selected the tags with B were included in the control group (CG, *n* = 14). Additionally, one participant in the IG withdrew voluntarily, another participant in the IG withdrew because his condition worsened, and one participant in the CG also withdrew voluntarily. Finally, the study included 12 PLWHA in the IG (age: 50.8 ± 11.2; height: 169.4 ± 7.0, HRmax: 169.2 ± 11.2) and 13 in the CG (age: 51.1 ± 10.9; height: 168.9 ± 6.0; HRmax: 168.9 ± 10). All patients underwent clinical follow-ups at the Specialized HIV/AIDS Care Service. All participants received a detailed explanation of the protocol and provided informed consent before participating in the study. The study was approved by the Research Ethics Committee of Kangwon National University (KWNUIRB-2022-05-002-001) and complied with the specific resolution of the Clinical Research Information Service (KCT0008668, 01/08/2023).

### Measurements

#### Anthropometrics assessment

HR and blood pressure (BP) were measured using a multifunction electronic sphygmomanometer. Height was measured using a stadiometer with the participants barefoot. Weight, skeletal muscle, body mass index (BMI), body fat, body fat percent, bone mineral, waist circumference, waist-hip ratio, visceral fat level, basal metabolic rate were assessed using bioelectrical impedance analysis (BIA) using InBody (Inbody 470, InBody Co. Ltd., Seoul, Korea). The device uses electrical impedance, along with age, weight, height, and sex information, to generate results based on InBody 470 body composition data.

#### Saliva sample

Saliva was collected using 1 mL plastic Salivette® collection tubes (Sarstedt Inc., Nümbrecht, Germany). Participants were not allowed to eat or drink for at least 60 min before collecting saliva samples and were instructed to gargle for at least 10 min before collection. Samples were collected before and after the experiment, and 3 samples were collected at once, immediately frozen, and stored at − 80 °C until biochemical analysis was performed. Before analysis, the saliva sample was thawed on ice and centrifuged at 1000xg for 2 min at 20 °C using an Allegra X-30R centrifuge (Allegra X-30R centrifuge, Beckman Coulte, Inc., Germany). Salivary testosterone (sal-T), salivary cortisol (sal-C) and secretory immunoglobulin A (sIgA) levels in the saliva were measured by an enzyme-linked immunosorbent assay using an ABclonal Kit (ABclonal Biotechnology Co., Ltd, MA, USA).

#### Questionnaires

The following information was collected using a questionnaire: demographic characteristics, QoL, stress, physical self-efficacy, dietary habits, dietary behavior, and exercise motivation.

Individual demographic characteristics, including marital status, children, occupation, census register, education, monthly household income, infection duration, and stage, were collected using a structured questionnaire. The Korean version of the World Health Organization (WHO) Quality of Life-HIV Brief developed by the WHO in 2002 was used [34], which consists of six areas and a total of 31 questions. The stress questionnaire developed by the Centers for Disease Control in Korea for analysis of the National Health and Nutrition was used, which consists of 3 subfactors (exhaustion, depression, and anger) with a total of 20 items, and is composed of a 5-point Likert scale ranging from “strongly disagree” (1 point) to “strongly agree” (5 points). Physical self-efficacy comprised 22 questions as sub-factors of cognition of physical ability and confidence in physical self-expression [35]. Dietary habits were used to assess food intake behavior. It consists of 14 items on the number, regularity, amount, and duration of meals; time and reason for skipping a meal; amount of rice; number and time of overeating; number of times eating out; number and time of snacks; number of late-night snacks; and unbalanced eating. A dietary behavior questionnaire (the Dutch Eating Behavior Questionnaire) [36] was translated into Korean and used after verifying its validity and reliability. The questionnaire consisted of 33 questions divided into 3 categories (restrained eating, emotional eating, and external eating). The questionnaire related to exercise motivation was used; 23 questions were applied as sub-factors of motivation, external observation, external motivation, internal motivation, achievement, and pleasure. The questionnaire consisted of a 5-point Likert scale.

#### Physical fitness

The physical fitness test included muscle grip strength, explosive strength, reaction speed, body flexibility, and cardiorespiratory endurance. Before the test, the participants performed a ten-minute warm-up to fully stretch the joints, ligaments, and muscles to prevent strain. All measurement items were cross-tested; each item was tested twice, and the best results were recorded. One exception to this was the cardiorespiratory endurance test, which was the last item and was only measured once.

Muscle strength was assessed by grip strength (kg) measured using a digital dynamometer (TKK 5401 Grip-D, Takei, Niigata, Japan) and recorded to one decimal place. The explosive power was shown in real-time jump metrics (cm) assessed using the Mayfonk Athletics VERT Wearable Fitness Monitor (Mayfonk Inc., Fort Lauderdale, FL, USA). The results were recorded in two decimal places. The reaction speed was evaluated based on the reaction time (s), and the test tool was a reaction-time measuring machine. The flexibility was assessed while sitting forward (cm). Cardiopulmonary endurance was shown as physical efficiency index (PEI) and assessed using the 5-minute step box test (high:30.5 cm). After testing, HR was recorded 3 times (post 1-1.5 min (HR1), post 2-2.5 min (HR2), post 3-3.5 min (HR3)). The calculation method was as follows:

1. When task was completed within the specified time:
  $$PEI\,{\text{=}}\,\frac{{{\text{Time}}\, * \,{\text{100}}}}{{{\text{2}}\, * \,\left( {{\text{HR1}}\,{\text{+}}\,{\text{HR2}}\,{\text{+}}\,{\text{HR3}}} \right)}}$$

2) When task was not completed within the specified time:
  $$PEI\,{\text{=}}\,\frac{{{\text{Time}}\, * \,{\text{100}}}}{{\left( {5.5\, * \,{\text{HR1}}} \right)}}\,+\,0.22\, * \,\left( {{\text{300}}\, - \,{\text{Time}}} \right)$$

Time is the test duration, expressed in seconds.

### Statistical analysis

Statistical analysis was performed using SPSS 26.0 (version for MAC; Chicago, IL, USA). Means and standard deviations (mean ± standard deviation [M ± SD]) were calculated for all variables. Comparisons of body composition, physical fitness, immune response, QoL and stress before and after the experiments were performed using a two-way analysis of variance (ANOVA) with repeated measures (group × time). When a significant interaction effect was observed, a simple effect was further analyzed, and when significant main effects were observed, post hoc testing was performed using a paired t-test. Statistical significance was set at *p* <.05.

## Results

### Demographic characteristics

None of the participants were married, and 84.6% and 66.7% had no children in the CG and IG, respectively. Moreover, 30.8% and 41.7% of participants in the CG and IG groups had no occupation, respectively (Table 1). Simultaneously, more than 90% of the participants in the two groups had an income of less than $800. In both groups, more than 65% of the participants were from rural areas, and 30% of the participants had graduated from college or other institutions of higher education. The duration of infection was the longest (> 11 years) in both groups, accounting for 46.1% and 41.7% of cases, respectively. During the infection stage, none of the participants reached the severe symptom stage and more than 65% were asymptomatic.

**Table 1:** Descriptive statistical analysis of individual demographic characteristics

	Information	CG(*N* = 13) Mean ± SD/N (%)	IG(*N* = 12) Mean ± SD/N (%)
Baseline characteristics	Age (years)	51.1 ± 10.9	50.8 ± 11.2
	Height (cm)	168.9 ± 6.0	169.4 ± 7.0
	HRmax (bpm)	168.9 ± 10.9	169.2 ± 11.2
Marital status	Single	9 (69.2%)	8(66.7%)
	Married	-	-
	Widowed	1 (7.7%)	-
	Divorced	3 (23.1%)	4(33.3%)
Children	Yes	2 (15.4%)	4(33.3%)
	No	11 (84.6%)	8(66.7%)
Occupation	Part-time job	3 (23.1%)	2(16.7%)
	Public servants	4 (30.8%)	3(25.0%)
	Self-employed	2 (15.4%)	2(16.7%)
	Unemployed	4 (30.8%)	5(41.7%)
Census register	Rural	9 (69.2%)	8(66.7%)
	Urban	4 (30.8%)	4(33.3%)
Education	Middle school graduate or lower	4 (30.8%)	3(25.0%)
	High school graduate	5 (38.5%)	4(33.3%)
	College or higher	4 (30.8%)	5(41.7%)
Monthly household income	<$800	12 (92.3%))	11(91.7%)
	≥$800	1 (7.7%))	1(8.3%)
Duration of infection (years)	0–5	2 (15.4%)	3(25.0%)
	6–10	5 (38.5%)	4(33.3%)
	≥ 11	6 (46.1%)	5(41.7%)
Infection stage	Asymptomatic	9(69.2%)	8(66.7%)
	Mild symptoms	1 (7.7%)	1(8.3%)
	Advanced Symptoms	3 (23.1%)	3(25.0%)
	Severe symptoms	-	-

HRmax: maximum heart rate; N: number; M ± SD: mean ± standard deviation

### Anthropometrics

The BP, HR, and body composition data are shown in Table 2. No significant differences were observed after the training period compared with pre-exercise training in either group or between the groups post-training.

**Table 2:** Descriptive statistical analysis of anthropometrics

	CG (N=13)		IG(N=12)	
	pre(M ± SD)	post(M ± SD)	Pre(M ± SD)	Post(M ± SD)
SBP (mmHg)	127.46 ± 9.20	122.92 ± 6.55	121.67 ± 4.92	120.92 ± 10.52
DBP (mmHg)	79.08 ± 15.98	76.31 ± 12.01	75.8 ± 11.67	74.58 ± 13.48
HRR (bpm)	80.92 ± 12.48	78.31 ± 12.51	80.75 ± 10.81	86.33 ± 13.25
Weight (kg)	69.80 ± 14.91	69.94 ± 14.75	72.58 ± 15.03	70.65 ± 15.01
Skeletal muscle (kg)	28.25 ± 4.76	27.06 ± 5.08	27.64 ± 4.92	27.53 ± 5.28
BMI (kg/m2)	25.09 ± 4.50	24.49 ± 4.38	25.28 ± 4.44	23.82 ± 4.38
Body fat (kg)	21.89 ± 8.50	21.19 ± 7.59	22.78 ± 8.42	21.10 ± 7.70
Body fat percent (%)	29.25 ± 6.92	29.67 ± 5.19	30.57 ± 6.03	29.23 ± 5.95
Bone mineral(kg)	2.80 ± 0.40	2.70 ± 0.44	2.78 ± 0.42	2.75 ± 0.47
Waist circumference(cm)	92.63 ± 13.66	90.16 ± 13.02	92.90 ± 13.66	90.24 ± 13.23
Waist-hip ratio	0.95 ± 0.08	0.93 ± 0.07	0.95 ± 0.08	0.93 ± 0.07
Basal metabolic rate (kcal)	1,465.00 ± 164.00	1,422.92 ± 180.00	1,445.92 ± 172.00	1,440.25 ± 188.08

M ± SD: mean ± standard deviation; SBP: systolic blood pressure; DBP: diastolic blood pressure; HRR: heart rate at rest; BMI: body mass index

### Physical fitness

There were significant differences in the reaction speed, grip strength, and PEI after the training period in the IG (Table 3). Performance of reaction (*p* =.001), grip strength (L: *p* =.002, R: *P* =.03), and PEI significantly improved after training in the IG (*p* =.023).

**Table 3:** Descriptive statistical analysis of physical fitness

	CG(*N* = 13)			IG(*N* = 12)	
	Pre(M ± SD)		Post(M ± SD)	Pre(M ± SD)	Post(M ± SD)
Reaction time (s)	0.39 ± 0.03		0.42 ± 0.03	0.45 ± 0.03	0.38 ± 0.03**
Grip strength (N)	L 31.82 ± 6.25		31.84 ± 6.25	31.61 ± 6.47	35.24 ± 6.09**
	R 33.68 ± 8.56		32.75 ± 9.03	32.18 ± 9.18	34.89 ± 7.10*
Sitting forward flexion (cm)	-7.12 ± 9.20		-6.42 ± 8.90	-5.29 ± 7.65	-4.275 ± 6.38
Explosive power (cm)	24.77 ± 8.10		24.15 ± 10.03	25.58 ± 6.54	27.25 ± 7.84
PEI	55.44 ± 8.49		60.04 ± 7.22	54.73 ± 8.27	61.00 ± 8.36*

M ± SD: mean ± standard deviation. PEI: physical efficiency index *p < .05, **p < .01

### Saliva

There were significant differences in sal-T levels after the training period in the IG (*p* =.012, Table 4). Compared to pre-training levels, sal-T levels significantly increased after the intervention in the IG (*p* =.012). However, there were no significant differences in sal-C, the ratio of sal-T to sal-C, and sIgA between the CG and IG.

**Table 4:** Descriptive statistical analysis of salivary

	CG(N=13)		IG(N=12)	
	pre(M ± SD)	post(M ± SD)	Pre(M ± SD)	Post(M ± SD)
sal-T (pg/mL)	35.40 ± 24.37	40.83 ± 30.17	28.45 ± 22.66	46.67 ± 26.80*
sal-C (ng/mL)	2.06 ± 0.68	3.30 ± 2.20	2.04 ± 0.74	2.88 ± 1.72
sal-T / sal-C	21.93 ± 28.36	18.76 ± 21.35	20.16 ± 31.43	21.18 ± 17.02
SIgA (ng/mL)	108.50 ± 19.63	109.67 ± 19.05	109.39 ± 20.23	104.56 ± 18.93

M ± SD: mean ± standard deviation; *p <.05

Sal-T: salivary testosterone; sal-C salivary cortisol; SIgA: secretory immunoglobulin A

### Subjective changes after training

Among the sub-factors of QoL, the overall level was “normal”, indicating that QoL was not perceived as high (Table 5). However, there were significant differences in physical satisfaction after the training period in the IG (*p* =.001). The perceived physical satisfaction improved significantly. Perceived stress exhibited low indices, suggesting that the stress levels were perceived as psychologically low. Statistically significant differences in burnout were observed after training in the IG (*p* =.043). Physical self-confidence in physical self-efficacy changed after the training period in the IG (*p* =.045). There was significant increase in emotional eating after the training period in the IG (*p* =.001). This value was significantly higher than the post-training value in the CG (*p* =.013). However, there were no significant post-training differences in restrained and external eating between the groups. Perceived external display significantly decreased (*p* =.008) but fulfillment significantly improved (*p* =.047) after training in the IG.

**Table 5:** Descriptive statistical analysis of psychological variables

	Category	CG(N=13)		IG(N=12)	
		Pre(M ± SD)	Post(M ± SD)	Pre(M ± SD)	Post(M ± SD)
QoL	Physical satisfaction	3.17 ± 0.92	3.25 ± 0.88	2.83 ± 0.91	3.29 ± 0.92***
	Psychological well-being	2.77 ± 0.86	2.83 ± 0.88	2.92 ± 0.85	2.77 ± 0.75
	Independent satisfaction	3.10 ± 0.87	2.90 ± 0.80	2.96 ± 0.80	3.02 ± 0.73
	Social relational satisfaction	2.69 ± 0.90	2.69 ± 0.88	2.75 ± 0.89	2.83 ± 0.78
	Environmental satisfaction	2.73 ± 0.92	2.80 ± 0.82	2.86 ± 0.82	2.91 ± 0.91
	Spiritual satisfaction	2.69 ± 0.85	2.73 ± 0.83	2.69 ± 0.80	2.60 ± 0.80
Stress	Burnout	2.87 ± 0.95	3.02 ± 1.00	2.94 ± 1.00	3.11 ± 0.98*
	Depression	2.49 ± 0.97	2.54 ± 1.03	2.51 ± 1.05	2.53 ± 0.99
	Anger	2.27 ± 0.87	2.54 ± 1.13	2.46 ± 1.13	2.63 ± 1.08
Physical self-efficacy	Cognition of physical ability	2.68 ± 0.96	2.66 ± 0.92	2.63 ± 0.91	2.69 ± 0.9
	Physical self-confidence	2.76 ± 0.90	2.64 ± 0.96	2.61 ± 0.95	2.78 ± 0.93*
Dietary behavior	Restrained eating	3.19 ± 0.87	3.08 ± 0.88	3.01 ± 0.87	3.07 ± 0.80
	Emotional eating	2.33 ± 0.74	2.35 ± 0.87	2.18 ± 0.75	2.58 ± 0.81***#
	External eating	3.14 ± 0.86	3.02 ± 0.92	2.93 ± 0.91	3.01 ± 0.84
Exercise motivation	Amotivation	-	-	2.90 ± 0.71	2.87 ± 0.83
	External display	-	-	2.54 ± 0.83	2.27 ± 0.73**
	Extrinsic motivation	-	-	2.50 ± 0.83	2.46 ± 0.77
	Intrinsic motivation	-	-	3.15 ± 0.80	3.29 ± 0.87
	Fulfillment	-	-	2.50 ± 0.85	2.81 ± 1.04*
	Pleasure	-	-	2.61 ± 0.83	2.75 ± 0.94

M ± SD: mean ± standard deviation

*:compared with pre-training in IG; * *p* <.05, **p <.01, ***p <.001

#:compared with post-training CG; # *p* <.05

### Dietary habits

In the survey on eating habits, similar dietary habits were observed between the two conditions (Table 6). More than 90% of the CG and IG ate twice a day, and most of them (76.9% in the CG and 66.7% in the IG) skipped breakfast. The amount of food consumed per meal was moderate (69.2% in the CG and 66.7% in the IG). One person in each group was slightly full, whereas none of them were very full. In the IG survey on dietary regularity, the number of people who ate regularly was the largest (50%), followed by those who ate irregularly (25%). However, in the CG, most of them ate irregularly (38.5%), and fewer people ate regularly (25%). Most of the participants in the IG (91.7%) took approximately 10–20 min or more than 20 min at every meal. However, in the CG, most people took more than 20 min, and the number of those who took less than 10 min or 10–20 min were the same (30.8%). Regarding overeating, the most common pattern in both groups was that of overeating one to two times per week or not overeating at all (CG, 77%; IG, 83.4%); moreover, overeating occurred mostly at dinner. Most participants ate out 1–2 times a week in both groups (CG 46.2%; IG 66.7%). In the CG 30.78% reported not eating out, while in the IG 16.7% reported not eating out. The number of people who ate snacks 1–2 times a week was the largest in the CG (30.8%), and those who ate snacks once a day was the largest in the IG (33.3%). Most snacks were consumed between dinner and lunch. More than 50% of the participants in both groups reported that they did not eat late-night meals.

**Table 6:** Descriptive statistical analysis of individual dietary habits

	Sub-variable	CG N (%)	IG N (%)		Sub-variable	CG N (%)	IG N (%)
Number of meals (day)	1	-	1(8.3)	Number of overeating	3 times < (day)	-	-
	2	12(92.3)	11(91.7)		2 times (day)	1 (7.7)	-
	3	1(7.7)	-		1 time (day)	1 (7.7)	2(16.7)
	4	-	-		3–4 times (week)	1 (7.7)	-
Meal time	Very irregular	3 (23.1)	2(16.7)		1–2 times (week)	4 (30.8)	5(41.7)
	Irregular	5 (38.5)	3(25.0)		No overeating	6 (46.2)	5(41.7)
	Regular	4 (30.8)	6(50.0)	Overeating meal	Breakfast	-	-
	Very regular	1 (7.7)	1(8.3)		Lunch	1 (24.3)	3(42.9)
Amount of meal	Very scarce	1 (7.7)	1(8.3)		Dinner	6 (85.7)	9(57.1)
	Little lack	2 (15.4)	1(8.3)	Number of eating out	1–2 times (day)	1(7.7)	-
	Moderate	9 (69.2)	8(66.7)		3–4 times (week)	2 (15.4)	2(16.7)
	Slightly full	1 (7.7)	1(8.3)		1–2 times (week)	6 (46.2)	8(66.7)
	Very full	-	-		No eating out	4 (30.8)	2(16.7)
Time period of meal	< 10 min	4 (30.8)	1(8.3)	Number of snack	3 times < (day)	-	-
	10–20 min	4 (30.8)	6(50.0)		2 times (day)	1 (7.7)	-
	> 20 min	5 (38.5)	5(41.7)		1 time (day)	1 (7.7)	4(33.3)
Skip a meal	Breakfast	10 (76.9)	8(66.7)		5–6 times (week)	2 (15.4)	2(16.7)
	Lunch	2 (15.4)	2(16.7)		3–4 times (week)	2 (15.4)	2(16.7)
	Dinner	1 (7.7)	1(8.3)		1–2 times (week)	4 (30.8)	3(25.0)
	No skip	-	1(8.3)		No snack	3 (23.1)	1(8.3)
Reasons for skipping breakfast	No skipping	2 (15.4)	3(25.0)	Snack time	Breakfast ∼ lunch	1 (7.7)	1(8.3)
	Over sleeping	1 (7.7)	1(8.3)		Lunch ∼ dinner	5 (38.5)	6(50.0)
	No appetite	2 (15.4)	3(25.0)		After dinner	3 (23.1)	1(8.3)
	Indigestion	1 (7.7)	-		Anytime	4 (30.8)	4(33.3)
	No preparing breakfast	-	1(8.3)	Number of late-night meal	Every day	1 (7.7)	-
	Lose weight	1 (7.7)	1(8.3)		3–4 times (week)	1 (7.7)	1(8.3)
	Habitually	6 (46.2)	3(25.0)		1–2 times (week)	2 (15.4)	3(25.0)
Amount of rice	More than a bowl	2 (15.4)	1(8.3)		1–3 times (month)	2 (15.4)	2(16.7)
	A bowl	5 (38.5)	7(58.3)		No late-night meal	7 (53.9)	6(50.0)
	2/3 bowl	4 (30.8)	2(16.7)	Un-balanced feeding	Yes	5 (38.5)	6(50%)
	1/2 bowl	2 (15.4)	2(16.7)				
					No	8 (61.5)	6(50%)
	1/3 bowl	-	-				

## Discussion

To the best of our knowledge, this is the first study to use exercise and diet therapy interventions for PLWHA. This study aimed to analyze the effects of exercise and diet therapy on QoL, stress levels, diet behavior, body composition, physical fitness, and immune responses in PLWHA following appropriate treatment. The main finding of this study was that exercise and diet therapy interventions positively affected physical fitness and Sal-T scores.

The demographic characteristics of the participants were similar between the groups. It could not be analyzed centering on a specific generation, due to the age distribution of the participants was wide. However, a special finding was that no participant had a full-time job despite them being in the asymptomatic infection stage and having a higher education level compared to the general public. Additionally, most participants had a monthly income of less than 800 USD. These findings are in line with those of a previous study indicating that the monthly income level was lower in HIV-positive people than in HIV-negative people [37]. These results reflect that infected people live economically insufficient lives because they do not actively participate in socio-economic life. In South Korea, the main reasons for this are the lack of a social system for infected people and the social conception of infected people rather than the lack of individual efforts. In fact, 58% of HIV-infected people are employed and most of them work full-time in Canada [38].

It is generally believed that a certain amount of exercise can effectively change anthropometric markers; however, no significant changes in anthropometrics were observed in the present study. The reason for this may be that the change in anthropometric measurements is a long-term process; therefore, the 4-week intervention time was too short. A previous study indicated that a 12-week exercise intervention period is needed to significantly change body mass index, body fat percentage, and waist-to-hip ratio [39]. Bone density can be an important variable among anthropometric changes achieved through exercise training interventions in PLWHA.

ART stimulates the activity of osteoclasts to increase bone absorption while reducing the activity of osteoblasts to inhibit bone production, resulting in reduced bone mineral density [40]. Optimal nutrient intake and regular exercise reduce bone density loss, weaken the progression of HIV infection, and improve immune function in HIV-infected individuals [41, 42]. In this study, nutrition and exercise treatment for four weeks did not cause changes in bone density. Both aerobic and resistance exercises can have a positive effect on bone density. Exercise intensity and training duration are important for increasing bone density through aerobic exercise, and high-impact and long-duration endurance training is effective in increasing bone density [43, 44]. However, these studies were carried out on the general public, and few studies have been conducted on the effects of nutritional supplementation and prolonged exercise training on bone density in PLWHA. Therefore, further studies are required to develop proper nutrition and exercise training strategies to increase bone density in PLWHA.

Exercise has a clear promoting effect on auditory stimulation [45]. The auditory reaction time decreased in the IG, confirming that auditory response ability can be improved through moderate aerobic exercise training. Most investigations on exercise in PLWHA have reported an increase in muscle strength [46] and improvement in cardiopulmonary function [13, 47–50]. In the present study, grip strength significantly increased, proving that exercise was beneficial for improving muscle strength. PEI, which is an indicator of cardiopulmonary efficiency and aerobic capacity, was relatively weak in all participants, indicating poorer cardiopulmonary efficiency. Poor aerobic fitness contributes to cognitive decline in PLWHA [51]. HIV infection causes various diseases and a considerable amount of pain, which may lead participants to adopt sedentary lifestyles [52] instead of exercising. The increase in PEI observed after the 4 weeks training period in this study confirmed the positive effect of regular exercise on PEI seen in previous studies [53–57]. These results are also supported by those of previous studies that analyzed the effects of exercise training on PLWHA. Regular exercise can improve cardiopulmonary function and efficiency in PLWHA without adverse effects [55, 56, 58, 59]. However, the results of endurance, flexibility, and explosive power did not change after training in this study. Based on the finding that different training loads and forms lead to different training adaptations [60], four weeks of moderate-intensity aerobic exercise is not an appropriate form and intensity to improve flexibility and power in PLWHA.

Saliva analysis has rapidly developed into a tool for evaluating physiological biomarkers commonly measured in the blood. Moreover, saliva collection without the stress caused by venipuncture can be performed quickly and frequently, reducing infectivity during sample collection. Salivary and serum measurements are perceived to be equivalent, and there are a wide variety of biomarkers in saliva, including immune and inflammatory factors. In this study, the ratios of sal-T, sal-C, and SIgA were analyzed to evaluate the effects of exercise training and dietary intervention on PLWHA. Sal-T, Sal-C, and their ratios have been reported as indicators of anabolic status [61], psychological stress, and exercise training effects [62–64]. After exercise training and diet intervention, the sal-T level in the IG increased significantly, which is consistent with previous research [64]. Exercise improves the therapeutic effects of testosterone treatment in PLWHA [65]. Although we did not analyze the relationship between the exercise-induced increase in sal-T and the therapeutic effect in this study, it can be inferred that the improvement of sal-T might be beneficial to the therapeutic procedure of PLWHA.

Cortisol is believed to be the primary hormone responsible for catabolic processes [66], reducing protein synthesis, increasing protein degradation, and inhibiting inflammatory processes and immunity [67]. Cortisol level monitoring during training has been reported as an approach to assess the impact of exercise training on the body’s catabolism and physical exertion [68]. Psychological stressors can induce an increase in salivary cortisol levels [69, 70], which is a good stress index [71]. The stress level of PLWHA was generally high [72]. Compared to the general population, the incidence of post-traumatic stress disorder is estimated to be quite high in PLWHA [73]. A certain amount of acute exercise can improve cortisol levels [74] but four weeks of moderate-intensity exercise did not affect the baseline cortisol levels in PLWHA. This result is consistent with that of a previous study that reported that baseline cortisol levels did not change after a long period of exercise training [75]. Therefore, to reduce the stress levels of PLWHA, various programs should be implemented in combination with exercise. The testosterone-to-cortisol ratio frequently counterbalances the catabolic state in PLWHA [64] and reflects the impact of exercise training on the overall recovery of the body [76]. However, there were no significant changes in cortisol levels or the testosterone-to-cortisol ratio in the present study. These results may be partly elicited by viral infections and the side effects of prolonged ART use. There may be an association between infection duration and ART, with the incidence causing a decrease in the anabolic state and consequently in muscle mass and function [77, 78] in PLWHA.

SIgA is considered the best indicator of mucosal immunity, as it acts as the first-line defense [79] and is considered the major antibody against upper respiratory tract infections [64]. Although a previous study has shown that exercise training and dietary intervention have a positive role in promoting the concentrations of sIgA in PLWHA [64, 80], the results of the present study showed no significant changes in sIgA levels after exercise training and dietary intervention for four weeks in PLWHA. The duration of aerobic exercise training in this study seemed insufficient to cause changes in IgA levels. Similar to the results of this study, another study showed that three weeks of training did not cause any changes in sIgA levels [81]; however, other studies showed a significant improvement in sIgA levels after 3–12 months of exercise training [82]. Taken together, more than four weeks of long-term exercise training is required to increase sIgA levels. Although we were unable to control the lifestyle of PLWHA during the study period, biological and lifestyle factors, such as life rhythm and caffeine intake, have been shown to affect sIgA levels [83].

Diet and exercise intervention for four weeks showed a significant improvement in physical satisfaction among the sub-factors of QoL. These results are in line with the results of a previous study that showed that exercise training had a positive effect on psychological well-being, independent satisfaction, and social-relational satisfaction [57]. In fact, most HIV/AIDS patients suffer from various problems that may be caused by PLWHA’s health level, fear of disease, and social pressure. Individuals’ awareness of their psychological and physical health may affect their QoL. Previous research has shown that a reduction in QoL is related to the negative psychological state and body health status of PLWHA [84]. Regular exercise improves QoL in PLWHA [85]; however, the perceived QoL may be related to the training period. Similar to QoL, the observation of significant improvement in physical self-expression and confidence in physical self-efficacy in this study supports this suggestion. Long-term regular exercise can improve the physical state in advance and have positive effects on psychological, social, and physical health, thus improving QoL. Indeed, a long period of regular exercise improved all the sub-values of QoL [86] and physical self-efficacy [87].

Stress levels can affect well-being. There was a significant increase in burnout in the IG after training. Burnout is mainly related to strain in daily life, which is often caused by the joint effects of long-term and high-intensity workloads, work environments, and conditions [88, 89]. Although there was no significant difference in depression and anger levels after training in the IG, the results showed an increase, indicating an overall increase in stress levels in the IG. This result does not support the widespread assumption that physical exercise is an effective means to reduce stress levels [90–92]. The training-induced increase in stress levels may be related to the participants’ physical fitness status, exercise, and the type of exercise performed. Most participants in this study did not perform regular physical activities, and their physical fitness was low. Owing to the low amount of physical activity and low fitness level at baseline, the exercise itself may have been stressful to the participants. In a previous study conducted in our laboratory, stress responses to acute treadmill exercises such as high cortisol levels were also observed in PLWHA [23]. The form of exercise performed can play an important role in increasing interest in exercise. Regardless of the participants’ will, the 45-minute moderate-intensity aerobic exercise determined and applied by the researchers may have been boring to the participants. Interval aerobic exercise was more enjoyable and had a greater effect than continuous aerobic exercise [93]. Exercise motivation and physical activity intention were higher in voluntarily selected training than in arbitrary training [94]. In line with these results, the external display was reduced after training, and there were no significant changes in most of the exercise motivation sub-variables in this study. Future studies on the efficacy of autonomy-based exercise training are required to analyze the effects of physical fitness and psychological components on PLWHA.

Regarding the psychometric properties of the participants’ dietary behavior test consisting of the three indicators of restrained, emotional, and external eating, only emotional eating significantly improved after the experiment in the IG. Emotional eating refers to eating stimulated by negative emotions, such as anger, fear, or anxiety [95, 96]. Training-induced influence on emotional eating may be attributed to complex factors. As mentioned earlier, exercise caused stress for participants with lower physical fitness and health status compared to the general public, and increased stress levels resulted in negative consequences for emotional eating. The participants had not exercised for a long time, and the sudden addition of regular exercise to their daily life affected the participants’ mental health, resulting in an increase in the level of stress. Stress caused by a lack of ability to cope with internal and environmental factors is closely related to emotional eating [97]. In addition, the increase in emotional eating scores may have been affected by the participants’ dietary habits. The IG reported that none of them ate regularly three times a day, and most of them ate breakfast twice because of poor appetite and habitual reasons. Physical activities such as preparing for work were not required in the morning, and unstable job conditions would lead to these dietary habits. Emotional eating may be increased through stress caused by the lack of ability to cope with stressful stimuli while performing exercise in a state of insufficient energy intake due to insufficient meals per day. A previous study has found a significant correlation between dietary energy intake and emotional eating [98].

This study has three major limitations. First, it was difficult to recruit participants owing to the characteristics of the study participants; therefore, the study group was composed of control, exercise, and diet nutrition intervention groups. We did not analyze the effects of exercise alone; therefore, it was not possible to determine the effect of nutritional supplementation during the training period compared to exercise alone. Second, the training period was four weeks, which was too short to verify the training effect. It is believed that the changes in psychological variables were not significant because of the short training period. Finally, a more scientific physical fitness test, such as gas analysis during treadmill running to analyze endurance and isokinetic muscle strength, was not conducted using a simple physical fitness test. Participants were extremely sensitive to identity exposure as PLWHA. Therefore, the tests were completed in the AIDS Prevention Association building, not in the laboratory, and there was a limit to the use of scientific test equipment. More scientific and reliable results could be obtained if an experiment is conducted to overcome these limitations in future research.

## Conclusion

In conclusion, a four-week combination of dietary supplementation and moderate-intensity exercise improved physical fitness and Sal-T scores in PLWHA. However, conflicting results have been obtained for psychological variables. There were some positive effects on QoL and physical self-efficacy; however, stress and daily behaviors were negatively affected. The negative effect on psychological variables may be related to the spontaneity of exercise participation. Therefore, it is necessary to implement a voluntary exercise program that can induce interest and persistence, along with proper dietary intake, to improve the health and well-being of PLWHA. An institutional support system should also be developed and provided by the government for PLWHA to participate in stable economic activities.

## Data Availability

The datasets generated during and/or analyzed during the current study are available from the corresponding author on reasonable request.

## Abbreviations

HIV: Human immunodeficiency virus
AIDS: Acquired immunodeficiency syndrome
PLWHA: People living with HIV/AIDS
QoL: Quality of life
IG: Intervention group
CG: Control group
PEI: Physical efficiency index
ART: Antiretroviral treatment
HR: Heart rate
THR: Target heart rate
DRS: Diet record sheet
BP: Blood pressure
BMI: Body mass index
BIA: Bioelectrical impedance analysis
Sal-T: Salivary testosterone
Sal-C: Salivary cortisol
SIgA: Secretory immunoglobulin A
WHO: World Health Organization
M ± SD: Mean ± standard deviation
ANOVA: Analysis of variance
L: Left
R: Right

## References

[1] Grant I, Atkinson JH, HESSELINK JR, KENNEDY CJ, RICHMAN DD, SPECTOR SA, McCUTCHAN JA (1987). Evidence for early central nervous system involvement in the acquired immunodeficiency syndrome (AIDS) and other human immunodeficiency virus (HIV) infections: studies with neuropsychologic testing and magnetic resonance imaging. Ann Intern Med.

[2] Sarkar D, Jung MK, Wang HJ (2015). Alcohol and the immune system. Alcohol Research: Current Reviews.

[3] World Health Organization, WHO. Global HIV, Programme. -HIV data and statistics. https://www.who.int/teams/global-hiv-hepatitis-and-stis-programmes/hiv/strategic-information/hiv-data-and-statistics

[4] Kim K, Kim S, Kim HS, Min S-Y. HIV/AIDS Notifications In Korea, 2022. 10.56786/PHWR.2023.16.46.2 PMC12480430 41333822

[5] Chun T-W, Moir S, Fauci AS (2015). HIV reservoirs as obstacles and opportunities for an HIV cure. Nat Immunol.

[6] Montessori V, Press N, Harris M, Akagi L, Montaner JS (2004). Adverse effects of antiretroviral therapy for HIV infection. CMAJ.

[7] Sayles JN, Hays RD, Sarkisian CA, Mahajan AP, Spritzer KL, Cunningham WE (2008). Development and psychometric assessment of a multidimensional measure of internalized HIV stigma in a sample of HIV-positive adults. AIDS Behav.

[8] Sayles JN, Wong MD, Kinsler JJ, Martins D, Cunningham WE (2009). The association of stigma with self-reported access to medical care and antiretroviral therapy adherence in persons living with HIV/AIDS. J Gen Intern Med.

[9] Kiecolt-Glaser JK, McGuire L, Robles TF, Glaser R (2002). Emotions, morbidity, and mortality: new perspectives from psychoneuroimmunology. Ann Rev Psychol.

[10] Segerstrom SC, Miller GE (2004). Psychological stress and the human immune system: a meta-analytic study of 30 years of inquiry. Psychol Bull.

[11] Amiri M, Hassani-Abharian P, Seyrafi MR. Designing a Community-Based Model of Adjustment Methods for Positive Prevention Based on Perceived Deterioration and Adherence Treatment Mediated by Coping Strategies in HIV-Positive Patients. *International Journal of Body, Mind & Culture (2345–5802)* 2021, 8(3).

[12] Smith BA, Neidig JL, Nickel JT, Mitchell GL, Para MF, Fass RJ (2001). Aerobic exercise: effects on parameters related to fatigue, dyspnea, weight and body composition in HIV-infected adults. Aids.

[13] Ozemek C, Erlandson KM, Jankowski CM (2020). Physical activity and exercise to improve cardiovascular health for adults living with HIV. Prog Cardiovasc Dis.

[14] Zourmand G, Pavlović R, Taheri M. The Effect of School games on Motor skills Development in Children with Autism. Annals of Applied Sport Science:0–0.

[15] Sheikhsaraf B, Peeri M, Azarbayjani M, Agha-Alinejad H (2016). The effect of aerobic interval training and massage therapy on c-reactive protein and cardiorespiratory fitness in cardiovascular patients after coronary artery bypass graft. Annals of Applied Sport Science.

[16] Simpson RJ, Kunz H, Agha N, Graff R (2015). Exercise and the regulation of immune functions. Prog Mol Biol Transl Sci.

[17] Esmailiyan M, Nobari H, Kargarfard M, Amerizadeh A, Esfarjani F, Vaseghi G, Badicu G, González PP, Ardigo LP. Effect of 12-Week Aerobic Exercise Training on Chemokine ligands and their relative receptors in Balb/C mice with breast Cancer. Int J Sport Stud Health 2022, 5(2).

[18] Dianatinasab M, Ghahri S, Dianatinasab A, Amanat S, Fararouei M. Effects of exercise on the immune function, quality of life, and mental health in HIV/AIDS individuals. Phys Exerc Hum Health 2020:411–21. 10.1007/978-981-15-1792-1_28 32342474

[19] Vader K, Simonik A, Ellis D, Kesbian D, Leung P, Jachyra P, Carusone SC, O’Brien KK (2017). Perceptions of ‘physical activity’and ‘exercise’among people living with HIV: a qualitative study. Int J Therapy Rehabilitation.

[20] Gebermariam BY, Naidoo R, Chetty V (2022). The effects of a 12-week exercise programme for people living with HIV in Ethiopia. Sport Sci Health.

[21] Freidenreich DJ, Volek JS. Immune responses to resistance exercise. Exerc Immunol Rev 2012, 18. 22876721

[22] Asimakos A, Toumpanakis D, Karatza M-H, Vasileiou S, Katsaounou P, Mastora Z, Vassilakopoulos T (2018). Immune cell response to strenuous resistive breathing: comparison with whole body exercise and the effects of antioxidants. Int J Chronic Obstr Pulm Dis.

[23] Qin X-M, Park J-Y, Kim B-R, Joo C-H (2022). The effects of exercise on acute immune responses in relative leisure-deprived people living with HIV/AIDS: a pilot study. Int J Environ Res Public Health.

[24] Kosmiski L (2011). Energy expenditure in HIV infection. Am J Clin Nutr.

[25] Organization WH. Living well with HIV/AIDS: a manual on nutritional care and support for people living with HIV/AIDS. In: *Living well with HIV/AIDS: a manual on nutritional care and support for people living with HIV/AIDS* edn.; 2012: 97–97.

[26] Wanke C, Silva M, Knox T, Forrester J, Speigelman D, Gorbach S (2000). Weight loss and wasting remain common complications in individuals infected with human immunodeficiency virus in the era of highly active antiretroviral therapy. Clin Infect Dis.

[27] Gambo A, Moodley I, Babashani M, Babalola TK, Gqaleni N. A double-blind, randomized controlled trial to examine the effect of Moringa oleifera leaf powder supplementation on the immune status and anthropometric parameters of adult HIV patients on antiretroviral therapy in a resource-limited setting. PLoS ONE 2021, 16(12 December). 10.1371/journal.pone.0261935 PMC8722362 34972169

[28] Singhato A, Khongkhon S, Rueangsri N, Booranasuksakul U (2020). Effectiveness of Medical Nutrition Therapy to Improve Dietary habits for promoting Bone Health in people living with chronic HIV. Ann Nutr Metab.

[29] HIV/AIDS JUNPo (2014). Nutrition Assessment, Counselling and support for adolescents and adults living with HIV: a PROGRAMMING GUIDE.

[30] Minnella EM, Awasthi R, Loiselle S-E, Agnihotram RV, Ferri LE, Carli F (2018). Effect of Exercise and Nutrition Prehabilitation on Functional Capacity in Esophagogastric Cancer surgery: a Randomized Clinical Trial. JAMA Surg.

[31] Taheri M, Irandoust K, Reynoso-Sánchez LF, Muñoz-Helú H, Cruz-Morales KN, Torres-Ramírez R, Mirmoezzi M, Youzbashi L, Mirakhori F, Dergaa I (2023). Effects of home confinement on physical activity, nutrition, and sleep quality during the COVID-19 outbreak in amateur and elite athletes. Front Nutr.

[32] Yagmaee F. Eight weeks of aerobic exercise and prescribed diet (low in carbohydrate and high protein) improve mental health in obese women. Int J Sport Stud Health 2021, 4(1).

[33] Ministry of Health and Welfare, MOHW. Nutrient intake status of Koreans based on nutrient intake standards. https://www.mohw.go.kr/board.es?mid=a10501010100&=0003

[34] World Health Organization, WHO. WHOQOL-HIV, bref. 2012 revision. https://apps.who.int/iris/handle/10665/77775

[35] Ryckman RM, Robbins MA, Thornton B, Cantrell P (1982). Development and validation of a physical self-efficacy scale. J Personal Soc Psychol.

[36] Van Strien T, Frijters JE, Bergers GP, Defares PB (1986). The Dutch eating Behavior Questionnaire (DEBQ) for assessment of restrained, emotional, and external eating behavior. Int J Eat Disord.

[37] Ogunmola OJ, Oladosu YO, Olamoyegun MA. Relationship between socioeconomic status and HIV infection in a rural tertiary health center. HIV/AIDS-Research and Palliative Care 2014:61–7. 10.2147/HIV.S59061 PMC4003148 24790469

[38] Worthington C, Krentz H (2005). Socio-economic factors and health-related quality of life in adults living with HIV. Int J STD AIDS.

[39] Gebermariam BY, Naidoo R, Chetty V. The effects of a 12-week exercise programme for people living with HIV in Ethiopia. Sport Sci Health 2022:1–9.

[40] McComsey GA, Tebas P, Shane E, Yin MT, Overton ET, Huang JS, Aldrovandi GM, Cardoso SW, Santana JL, Brown TT (2010). Bone disease in HIV infection: a practical review and recommendations for HIV care providers. Clin Infect Dis.

[41] Grobler L, Siegfried N, Visser ME, Mahlungulu SS, Volmink J. Nutritional interventions for reducing morbidity and mortality in people with HIV. Cochrane Database of Systematic Reviews 2013(2). 10.1002/14651858.CD004536.pub3 23450554

[42] Santos WR, Santos WR, Paes PP, Ferreira-Silva IA, Santos AP, Vercese N, Machado DR, de Paula FJA, Donadi EA, Navarro AM (2015). Impact of strength training on bone mineral density in patients infected with HIV exhibiting lipodystrophy. J Strength Conditioning Res.

[43] Chilibeck PD, Sale DG, Webber CE (1995). Exercise and bone mineral density. Sports Med.

[44] Berro AJ, Kazwini S, Ahmaidi S, Hage RE. Effects of 12 months of resistance training vs. endurance training on bone mineral density, hip geometry indices and trabecular bone score in a group of young overweight women. 2020.

[45] Yagi Y, Coburn KL, Estes KM, Arruda JE (1999). Effects of aerobic exercise and gender on visual and auditory P300, reaction time, and accuracy. Eur J Appl Physiol Occup Physiol.

[46] O’Brien KK, Davis AM, Chan Carusone S, Avery L, Tang A, Solomon P, Aubry R, Zobeiry M, Ilic I, Pandovski Z (2021). Examining the impact of a community-based exercise intervention on cardiorespiratory fitness, cardiovascular health, strength, flexibility and physical activity among adults living with HIV: a three-phased intervention study. PLoS ONE.

[47] Rigsby LW, Dishman R, Jackson AW, Maclean G, Raven P. Effects of exercise training on men seropositive for the human immunodeficiency virus-1. Medicine & Science in Sports & Exercise; 1992. 1548998

[48] Roubenoff R, McDermott A, Weiss L, Suri J, Wood M, Bloch R, Gorbach S (1999). Short-term progressive resistance training increases strength and lean body mass in adults infected with human immunodeficiency virus. Aids.

[49] Spence D, Galantino M, Mossberg K, Zimmerman S (1990). Progressive resistance exercise: effect on muscle function and anthropometry of a select AIDS population. Arch Phys Med Rehabil.

[50] Ibeneme S, Omeje C, Myezwa H, Ezeofor SN, Anieto E, Irem F, Nnamani AO, Ezenwankwo FE, Ibeneme G (2019). Effects of physical exercises on inflammatory biomarkers and cardiopulmonary function in patients living with HIV: a systematic review with meta-analysis. BMC Infect Dis.

[51] Mapstone M, Hilton TN, Yang H, Guido JJ, Luque AE, Hall WJ, Dewhurst S, Shah K (2013). Poor aerobic fitness may contribute to cognitive decline in HIV-infected older adults. Aging and Disease.

[52] Tegene Y, Mengesha S, van der Starre C, Lako S, Toma A, Spigt M (2022). Physical activity level and associated factors among adult HIV patients in Ethiopia. BMC Infect Dis.

[53] Briggs BC, Ryan AS, Sorkin JD, Oursler KK (2021). Feasibility and effects of high-intensity interval training in older adults living with HIV. J Sports Sci.

[54] Oursler KK, Sorkin JD, Ryan AS, Katzel LI (2018). A pilot randomized aerobic exercise trial in older HIV-infected men: insights into strategies for successful aging with HIV. PLoS ONE.

[55] Jaggers JR, Hand GA (2016). Health benefits of exercise for people living with HIV: a review of the literature. Am J Lifestyle Med.

[56] Odunaiya NA, Agbaje SA, Adegoke OM, Oguntibeju OO (2022). Effects of a four-week aerobic exercise programme on depression, anxiety and general self-efficacy in people living with HIV on highly active anti-retroviral therapy. AIDS Care.

[57] Oliveira VHF, Rosa FT, Santos JC, Wiechmann SL, Narciso AMS, de Moraes SMF, Webel AR, Deminice R (2020). Effects of a Combined Exercise Training Program on Health Indicators and Quality of Life of People Living with HIV: a Randomized Clinical Trial. AIDS Behav.

[58] Gomes Neto M, Conceicao CS, Carvalho VO, Brites C (2015). Effects of combined aerobic and resistance exercise on exercise capacity, muscle strength and quality of life in HIV-infected patients: a systematic review and meta-analysis. PLoS ONE.

[59] O’Brien KK, Tynan AM, Nixon SA et al. Effectiveness of aerobic exercise for adults living with HIV: systematic review and meta-analysis using the Cochrane collaboration protocol. BMC Infect Dis 2016, 16(182). 10.1186/s12879-016-1478-2 PMC4845358 27112335

[60] Kawamori N, Haff GG (2004). The optimal training load for the development of muscular power. J Strength Conditioning Res.

[61] Cook CJ, Kilduff LP, Beaven CM (2014). Improving strength and power in trained athletes with 3 weeks of occlusion training. Int J Sports Physiol Perform.

[62] Kilduff L, Cook CJ, Bennett M, Crewther B, Bracken RM, Manning J (2013). Right–left digit ratio (2D: 4D) predicts free testosterone levels associated with a physical challenge. J Sports Sci.

[63] Cook CJ, Kilduff LP, Crewther BT, Beaven M, West DJ (2014). Morning based strength training improves afternoon physical performance in rugby union players. J Sci Med Sport.

[64] Melo BP, Guariglia DA, Pedro RE, Bertolini DA, de Paula Ramos S, Peres SB, de Moraes SMF (2019). Combined exercise modulates cortisol, testosterone, and immunoglobulin A levels in individuals living with HIV/AIDS. J Phys Activity Health.

[65] Wagner G, Rabkin J, Rabkin R (1998). Exercise as a mediator of psychological and nutritional effects of testosterone therapy in HIV + men. Med Sci Sports Exerc.

[66] Viru A, Viru M (2004). Cortisol-essential adaptation hormone in exercise. Int J Sports Med.

[67] Sapolsky RM, Romero LM, Munck AU (2000). How do glucocorticoids influence stress responses? Integrating permissive, suppressive, stimulatory, and preparative actions. Endocr Rev.

[68] Papacosta E, Nassis GP (2011). Saliva as a tool for monitoring steroid, peptide and immune markers in sport and exercise science. J Sci Med Sport.

[69] Hill C, Walker R (2001). Salivary cortisol determinations and self-rating scales in the assessment of stress in patients undergoing the extraction of wisdom teeth. Br Dent J.

[70] Miller CS, Dembo JB, Falace DA, Kaplan AL (1995). Salivary cortisol response to dental treatment of varying stress. Oral Surg Oral Med Oral Pathol Oral Radiol Endodontology.

[71] Takai N, Yamaguchi M, Aragaki T, Eto K, Uchihashi K, Nishikawa Y (2004). Effect of psychological stress on the salivary cortisol and amylase levels in healthy young adults. Arch Oral Biol.

[72] Feng M-C, Feng J-Y, Yu C-T, Chen L-H, Yang P-H, Shih C-C, Lu P-L (2015). Stress, needs, and quality of life of people living with human immunodeficiency virus/AIDS in Taiwan. Kaohsiung J Med Sci.

[73] Ayano G, Duko B, Bedaso A (2020). The prevalence of post-traumatic stress disorder among people living with HIV/AIDS: a systematic review and meta-analysis. Psychiatr Q.

[74] Hill E, Zack E, Battaglini C, Viru M, Viru A, Hackney A (2008). Exercise and circulating cortisol levels: the intensity threshold effect. J Endocrinol Investig.

[75] Nazari M, Shabani R, Dalili S (2020). The effect of concurrent resistance-aerobic training on serum cortisol level, anxiety, and quality of life in pediatric type 1 diabetes. J Pediatr Endocrinol Metab.

[76] Terburg D, Morgan B, van Honk J (2009). The testosterone–cortisol ratio: a hormonal marker for proneness to social aggression. Int J Law Psychiatry.

[77] Cohen S, Nathan JA, Goldberg AL (2015). Muscle wasting in disease: molecular mechanisms and promising therapies. Nat Rev Drug Discovery.

[78] Morley JE, Thomas DR, Wilson M-MG (2006). Cachexia: pathophysiology and clinical relevance. Am J Clin Nutr.

[79] Mazanec MB, Nedrud JG, Kaetzel CS, Lamm ME (1993). A three-tiered view of the role of IgA in mucosal defense. Immunol Today.

[80] Gleeson M, McDONALD WA, Pyne DB, Cripps AW, Francis JL, Fricker PA, Clancy RL (1999). Salivary IgA levels and infection risk in elite swimmers. Med Sci Sports Exerc.

[81] Tiollier E, Gomez-Merino D, Burnat P, Jouanin J-C, Bourrilhon C, Filaire E, Guezennec C, Chennaoui M (2005). Intense training: mucosal immunity and incidence of respiratory infections. Eur J Appl Physiol.

[82] Kimura F, Shimizu K, Akama T, Akimoto T, Kuno S, Kono I (2006). The effects of walking exercise training on immune response in elderly subjects. Int J Sport Health Sci.

[83] Pritchard BT, Stanton W, Lord R, Petocz P, Pepping G-J (2017). Factors affecting measurement of salivary cortisol and secretory immunoglobulin A in field studies of athletes. Front Endocrinol.

[84] Zeluf-Andersson G, Eriksson LE, Schönnesson LN, Höijer J, Månehall P, Ekström AM (2019). Beyond viral suppression: the quality of life of people living with HIV in Sweden. AIDS Care.

[85] Ciccolo JT, Jowers EM, Bartholomew JB (2004). The benefits of exercise training for quality of life in HIV/AIDS in the post-HAART era. Sports Med.

[86] Arslan SS, Alemdaroğlu İ, Karaduman AA, Yilmaz ÖT (2019). The effects of physical activity on sleep quality, job satisfaction, and quality of life in office workers. Work.

[87] Kim S, Park W (2019). The effect of the elderly exercise program using elastic-band on the depression and physical self-efficacy of the elderly. J Korean Soc Integr Med.

[88] Naczenski LM, de Vries JD, van Hooff ML, Kompier MA (2017). Systematic review of the association between physical activity and burnout. J Occup Health.

[89] Ekstedt M, Fagerberg I (2005). Lived experiences of the time preceding burnout. J Adv Nurs.

[90] Galper DI, Trivedi MH, Barlow CE, Dunn AL, Kampert JB (2006). Inverse association between physical inactivity and mental health in men and women. Med Sci Sports Exerc.

[91] Bandelow B, Bayas A, Broocks A, Busse O, Geese R, Heuß D, Holzgraefe M, Junghanns K, Koch A, Kornhuber J, Neurologie. Psychiatrie und Sport: Georg Thieme Verlag; 2003.

[92] Bretland RJ, Thorsteinsson EB (2015). Reducing workplace burnout: the relative benefits of cardiovascular and resistance exercise. PeerJ.

[93] Bartlett JD, Close GL, MacLaren DP, Gregson W, Drust B, Morton JP (2011). High-intensity interval running is perceived to be more enjoyable than moderate-intensity continuous exercise: implications for exercise adherence. J Sports Sci.

[94] Sfandyari B, Ghorbani S, Rezaeeshirazi R, Noohpisheh S (2020). The effectiveness of an autonomy-based exercise training on intrinsic motivation, physical activity intention, and health-related fitness of sedentary students in middle school. Int J School Health.

[95] Kaplan HI, Kaplan HS (1957). The psychosomatic concept of obesity. J Nerv Ment Dis.

[96] Reichenberger J, Schnepper R, Arend A-K, Blechert J. Emotional eating in healthy individuals and patients with an eating disorder: evidence from psychometric, experimental and naturalistic studies. *Proceedings of the Nutrition Society* 2020, 79(3):290–299. 10.1017/S0029665120007004 PMC7663318 32398186

[97] Tan CC, Chow CM (2014). Stress and emotional eating: the mediating role of eating dysregulation. Pers Indiv Differ.

[98] Rasouli A, Moludi J, Foroumandi E, Shahsavari S, Ebrahimi B (2019). Emotional eating in relation to anthropometric indices and dietary energy intake based on gender. Mediterranean J Nutr Metabolism.

